# The Medical Year 1874

**Published:** 1875-03

**Authors:** 


### Selections.

### THE MEDICAL YEAR 1874.

[From the London Lancet.]

Medicine is not only a year older since our last summary, but a year wiser and a year more effective in its knowledge of disease and of methods for controlling it.

In Physiology, one of the principal works that have been published since the close of the last year, is Dr. Klein's memoir on the Lymphatic System, in which the distribution of the lymphatics, as well as the arrangement of the epithelium on the serous membranes, is very fully considered ; the general result arrived at being that the serous membranes are to be regarded as unraveled lymphatic glands, and, as such, play an important part in the formation of the white corpuscles of the blood. Drs. Foster and Balfour have just published a work that has long been needed—"The Elements of Embryology"—in which the process of development is described, in a singularly lucid and intelligible manner, from the earliest period in the fowl, and which constitutes the first part of a larger work, on the same subject, embracing the history of development in other animals. Dr. B. has also done a good piece of work in following the development of the dog-fish and its allies.

Mr. Parker has continued his elaborate account of the development of the skull, in his lectures delivered at the Royal College of Surgeons. Arnold, Gasser, Rauber, Mihalkovics, Sernoff, von Torok, Merkel, and many others, have devoted themselves to the elucidation of the process of development of various special parts or organs, as the eye, ear, pituitary body, testis, etc. Mr. St. George Mivart has published a brief but useful account of the Anatomy of the Frog. Quite recently, Mr. Barker has published a valuable translation of Frey's Histology. A very interesting little work has been issued in the International Scientific Series, by M. Marey, on "Animal Mechanism and Aerial Locomotion," in which great ingenuity is displayed in the application of recording apparatus to the registration of the movements of animals, both in aerial and in terrestrial locomotion. To the same series Dr. Draper, of New York, has contributed a volume that will be read with interest, on the "History of the Conflict between Religion and Science," in which the learned author, who is well known for his excellent treatise on Human Physiology, has endeavored to present a clear and impartial statement of the views and acts of the two contending parties. There is also a third, consisting of a translation of Oscar Schmidt's doctrine of descent and Darwinism. A popular physiology is contained in "Hinton's Physiology for Practical Use." A complete account of the Physiology of Food and Dietetics has been written by Dr. Pavy. Dr. Carpenter has developed two or three chapters on Mental Physiology contained in the earlier editions of his Physiology, which have been excluded from the later ones for want of space, into a goodly volume, written with his usual clearness and intelligence.

Amongst foreign works may be mentioned the "History of Creation" and the "Development of Man," by Haeckel, the former of which has been translated into English by Lankester, and the latter into French. Herrman has published a fifth edition of his excellent work on Physiology, which is now, we believe, in course of translation into English. Dr. Gustav Le Bon, in a large volume entitled "La Vie," remarkably well illustrated, and M. Coyteux, in another, the "Études sur la Physiologie," have both treated fully of human physiology. Hitzig has collected his observations and inquiries into the functions of the brain into one volume, which is worthy of careful perusal. Though, perhaps, scarcely capable of being included under the head of Anatomy and Physiology, we must not omit to chronicle the first International Congress of Orientalists, which was held at the Royal Institution in London, in September, and which under the presidentship of Dr. Birch, was the occasion of several excellent addresses, amongst which those of Max Muller, the President, and Prof. Owen, deserve special mention. Prof. Rutherford has been appointed to the Edinburgh Chair of Physiology, and carries with him the good wishes of his late colleagues in King's College, and of his friends in London. We trust that still increasing numbers of students in attendance upon his classes will testify to the ability with which he conducts them. A large number of separate contributions have been made up by individual observers, amongst which it is difficult to select any of pre-eminent value. Braun has repeated Hitzig's experiments on the excitability of the surface of the cerebrum by weak electrical currents, and whilst generally confirming his experiments, and those of Ferrier, adds this novel point, that there are several cases in which there appear to be two centres on the surface of the hemispheres for the same set of muscles, as, for instance, those of the neck. He also finds that, after shaving off the gray matter from a given spot, the groups of muscles can be made to contract, if the current be applied to the cut surface of the white substance as before, showing that the nerve-fibres really arise from the gray matter, whilst, if the white substance be divided, all manifestation of nervous excitation ceases. Burdon Sanderson has also made experiments upon this point, and has shown that there are points in the corpus striatum of the same side which correspond to those of the same hemisphere. Dr. Ferrier considers that in Dr. Sanderson's experiments only a medullary corresponding to the cortical centre is removed. Nothnagel, from experiments on the thalami optici, has come to the conclusion that they have no relation to voluntary muscular movements, nor to the general sensibility of the skin, but that they are immediately connected with the muscular sense. It is pleasant and encouraging, in connection with the subject of Physiology, to call to mind the meeting of the Russian Association for the Advancement of the Natural Sciences, held at Kasan, a flourishing city in the very centre of European Russia, on the Volga, which possesses a good university. Physiological subjects figured largely in the discussions. We may mention especially Dr. A. Schakowsky's paper on the Amount of Fat in Human Milk, averaging 3 per cent., and the striking reduction in the amount produced by a strictly vegetable diet, till it fell to 0.8 per cent.; Prof. Kowalewsky's paper on the Mechanics of the Movements of the Bile; and the paper of Dr. A. Troitzky, of Kasan, on a method of estimating the rapidity with which a stimulus is propagated under different temperatures, and with various strength of exciting currents. The maximum rapidity of propagation of stimulus is between 68° and 50° F.

The experience of the year has shown that Esmarch's plan of preventing hæmorrhage is capable of general application with advantage, and it has been almost universally adopted. Dittel's elastic ligature for the treatment of fistula in ano has been lately highly praised by Mr. Allingham, who has treated a number of cases with it satisfactorily. An interesting correspondence took place in our columns in January as to the credit of priority in the use of the elastic bandage and of the elastic ligature. According to our correspondent, Dr. Gesualdo Clementi, of Naples, Dr. Grandesso Silvestri, of Vicenza, used the elastic ligature in 1862; and he and Professor Vanzetti, of Padua, together used and described the elastic bandage very much in the same way as Esmarch. The subject of bloodless surgery, by means of the galvanic cautery, has been discussed at length by Dr. Bryant, and the introduction of a new and constant battery with carbon and bichromate of potash for its elements, seems to remove one of the difficulties hitherto in the way of the general adoption of the plan. In connection with the subject of Pyæmia, we may refer to Mr. Lister's practice, as reported upon by Mr. Gamgee, which serves to illustrate the proverbially diverse effect produced by the same series of phenomena in different minds. The treatment of Syphilis was the subject of an address to the Hunterian Society by Mr. Jonathan Hutchinson, who proved, we imagine, to the satisfaction of all candid inquirers, that mercury is the remedy for that loathsome disorder. The subcutaneous injection of mercury was the subject of an interesting communication by Mr. Cullingworth, but the method is one which has hitherto found little favor in this country. Sir William Fergusson has introduced a modification of the method of operating on cleft hard palate, which seems to promise well; and the same eminent surgeon was able to relieve public and private anxiety as to the identity of Livingstone's remains by the recognition of the united fracture of the humerus. Mr. Teale has called attention to the operation of ovariotomy in exceptional cases; Mr. John Wood has contributed to the literature of thoracentesis, and Mr. Reginald Harrison to that of puncture of the bladder by the aspirator. The recent lecture on American Surgery by Mr. Erichsen has set before us many points of interest in connection with the surgery and hospitals of the United States; and Dr. Buchanan's introductory address served to illustrate the activity of our northern brethren. Amongst the feats of surgery for the year, we may mention Billroth's total extirpation of the larynx and epiglottis for carcinoma—the second operation of the kind which he has performed; also his second complete removal of the thyroid gland, and the extirpation of a fibro-cystic tumor of the uterus, weighing sixteen pounds, with the uterus and its appendages, followed by recovery, by Dr. E. H. Trenholme, of Montreal. The "Lancet" of July 25th, sets forth that Dr. Henry Harland, of Wadhurst, Sussex, had extricated a Waterloo bullet from the hand of James Jenner, aged eighty-three. The said bullet had been in the veteran's hand fifty-nine years, and had become inconvenient to him at his work as a gardener! Our pages have contained many illustrations of the happy use of the aspirator in tapping the pericardium and other cavities, and Dr. Maclean speaks of the pus let out of livers at Netley by this instrument in a summer as measuring hundreds of ounces.

Of papers of importance at the Medico-Chirurgical Society, those by Dr. Cunningham on Recent Experience of Cholera in India, and by Surgeon A. Hall on the use of Chloral subcutaneously injected in that disease; by Mr. Mahomed on the Prealbuminuric Stage of Bright's Disease; by Dr. George Johnson on the Laryngeal Symptoms produced by Pressure on the Vagus and Recurrent Nerves,—may be noted. Much valuable matter has been brought forward at the Medical Society, which shows no signs of decay; and we must not omit to mention the able Lettsomian lectures delivered before the Society by Dr. Broadbent, on the subject of Syphilitic Affections of the Nervous System. The most important papers at the Obstetrical Society have been those of Puerperal Thrombosis, by Dr. Playfair, and on the Relation of Flexion and Congestion of the Uterus, by Dr. John Williams. We may note also a discussion at a special meeting on the admission of ladies as members of the society, which resulted in a decision adverse to the proposal.

The summer months brought the usual pleasant gatherings of scientific societies, in which not a few of our brethren seem to find their holiday. The meeting of the British Medical Association at Norwich was large and successful. The addresses were of a high character, and the Society of the old city was full of worth and hospitality. Amongst the distinguished foreigners was M. Magnan, who assayed to illustrate on dogs the different action of alcohol and absinthe, and in so doing involved some of the spectators in a criminal prosecution, at the instigation of the Society for the Prevention of Cruelty to Animals.

The meeting of the British Association at Belfast was presided over by Professor Tyndall, who lauded matter in such terms as to offend the sensitive religious minds of the Irish. He indicated that he saw in matter promise and the potency of all things. It is only fair to add that he has since confessed that when he looks abroad on the beauty of the universe, he cannot resist the feeling that there must be some Being superior to it and more familiar with it than he himself. And so we think, We have great regard for Professor Tyndall and his colleagues of the British Association; but we trust that there is a Being somewhere in the wide universe to whom even the knowledge of our wisest *savans* appears very elementary. Professor Huxley's address in Physiology on the Hypothesis that Animals are Automata, and Dr. Hooker's on the Carnivorous Habits of Plants, Professor Redfren's paper on the Effects of Ozone on the Animal Economy, Professor Cleland's on the Morphology of the Brain and the Function of Hearing, Sir Duncan Gibb's case of an old woman of 111, and other communications, are full of scientific or human interest. The Social Science Congress met in Glasgow. One of the best features of the meeting was a very able address from the chair of the Public Health section by Dr. Lyon Playfair. During the sitting of the British Association, congratulation and friendly sentiments were exchanged with the French Association for the Advancement of Science which met at Lille. The Medical section of the French meeting was well attended, and the scientific work of it was good. The Public Analysts are now becoming an important power in the community. They held a meeting at Cannon-Street Hotel, on Friday, August 7th, and in several resolutions showed their mind on questions involved in the working of the Adulteration Act.

The General Medical Council had a long and important sitting in the very hot days of July, from the 9th to the 18th. The business was opened by a very perfect address from Dr. Paget, the retiring President. In the pith of the report of the visitors there is claimed a terrible defect in preliminary education.

Several cases of rheumatic fever have been published, in which almost every conceivable remedy has been given with varying success. The acid treatment and the alkaline treatment, quinine, iron, and propylamin, have all been shown to be valuable, or at least not hurtful, in some cases of acute articular rheumatism. The value, in epilepsy, of bromide of potassium in large doses frequently repeated, was illustrated in a patient under the care of Dr. Ransome, of the Nottingham General Hospital. From Charing-cross Hospital we published a case showing the value of chloroform in chorea, and from the Royal United Hospital, Bath, another showing the value of chloral hydrate in the same affection. Lastly, we lately recorded a case from the University College Hospital, in which a child was shown to have suffered from an acne-like skin eruption, shrewdly traced by Dr. Tilbury Fox to taking milk from its mother, who was under the influence of bromide of potassium.

In Therapeutics, interest still centres to a very large extent in substances which, as a matter of fact, affect the nervous system, such as alcohol, chloral, belladonna, physostigma, phosphorus, etc. The value of alcohol as a therapeutic agent is still a much contested point. But we have lately had to remark on the great care and moderation with which it is prescribed by the present leaders of medical practice. The care with which a spirit ration was served out, and its effects watched, in the Ashantee war, made the subject very interesting. To say the least, the teetotalers sustained in that warfare a creditable amount of health, and in sickness showed a creditable amount of recuperative power. The Gold Coast campaign may be held to have proved, in the words of Dr. Parkes, that the rum ration should not be given in greater quantities than two ounces and a half per man daily, and that the time for giving it is not before or during, but after, a march. We may hope that in the future the spirit ration will be, as it was here, an extra to be given when deemed expedient by those best able to judge of circumstances and the wants of men. That remarkable medicine, physostigma, stands credited, on the testimony of Dr. Crichton Browne, at the Medical Society of London, with having cured two cases of general paralysis. Supposing there to have been no error of diagnosis, and no misconstruction of the relation of events in these cases, we should say that this is the most remarkable therapeutical achievement recorded in the year—much more remarkable than the several cases of tetanus apparently cured by chloral. The application of the curative powers of this latter medicine become multiplied. Unfortunately the use of the drug as a tippling agent also increases. Several cases of poisoning by it have occurred. Amongst the purposes for which it is recommended, with some show of reason, is as an anæsthetic in labor, and as a remedy, hypodermically injected, in cholera. In two or three cases of exophthalmic goitre, under the care of Dr. R. T. Smith, very great relief and advantage were obtained by the employment of belladonna. The question of the use of the cold bath in typhoid fever has been raised by an amateur therapeutist in the "Times;" but there has been no important addition to our knowledge of its value. In the serious epidemic of typhoid at Lyons it was used extensively, but with rather injurious results in the production of congestion, etc., and a correspondingly high rate of mortality. The bath used seemed to be at too low a temperature—namely, 68° F., and the use of it was not sufficiently restricted to cases of hyperpyrexia. In November, Dr. Clifford Albutt, of Leeds, discussed the mode of death in the early days of scarlatina, and gave proof—first, of the hyperpyrexia as a cause of death; secondly, of the efficiency of cool bathing in averting the tendency to death. The use of ipecacuanha in the form of spray for cases of winter cough and bronchitic asthma is considered by Dr. Ringer.

Dr. Spencer Cobbold has just finished an account of cases illustrative of the treatment of tapeworm, in which he shows at once the Protean effects of this intestinal guest, and the efficacy of discriminate treatment. He evidently revels in the possession of nineteen tapeworm heads from eighteen patients, not only as the guarantee of complete cure, but as a rich accession to his cabinet specimens.

Although the closing year is not likely to be remembered as one in which crime was less in amount than usual, there has, nevertheless, been an absence of crime of a sensational order. Public attention has not been roused by any villainous or subtle atrocity, and medical jurists have not been called upon to unravel any startling mysteries. The great question of identity involved in the Tichborne trial has been settled, and perhaps we may go so far as to say that the Lord Chief Justice has originated a new principle of identification by his axiom that however closely two persons may resemble each other anatomically, yet "no two persons ever were alike within." Certain it is that his masterly dissection of the inward Orton, and his comparison of it with the inward Tichborne, served to carry conviction, when scars, thumbs, ears, twitchings, brown marks, and peculiarities had merely tended to make confusion worse confounded. One of the most important advances in forensic medicine has been made by Dr. Richardson, of Philadelphia, who, by applying a micrometer and the highest powers of the microscope, has shown that a distinctly recognizable difference exists in the size of the blood-cells of man and the other mammalia.

The science of toxicology is threatened with a severe shock from an unexpected source.. The practice of cremation has been strongly advocated, and should it come into vogue there will be, of course, an end to "exhumation by order of the coroner." Civil cases, involving medical questions have not been numerous. Payment of a life policy has been refused on the ground that material information as to the habits of the assured was withheld, and although a verdict was originally obtained for the plaintiff, the insurance company obtained a rule for a new trial. The case of Simpson vs. Davey was one of a peculiar and distressing nature, and has roused the sympathy of the whole profession. Dr. Davey has had to pay £500 damages (besides law expenses) because, having unfortunately contracted syphilis at a midwifery case, and having affected with it another patient (Mrs. Simpson), and, further having undertaken to treat Mrs. Simpson free of charge till she was well, he failed to fulfill his contract. We would particularly remind our readers that Dr. Davey has suffered, not on the ground of having infected a patient, but merely on the ordinary ground of non-fulfillment of a contract.

The year will be remarkable in epidemiological annals, first, for the reappearance of plague in no less than three different localities of Asia and Africa; and, next (we may more reasonably hope), for the cessation of the long-continued prevalence of cholera in Europe. Early in the present year plague broke out in Mesopotamia, on the lower Euphrates; somewhat later it appeared in the Regency of Tripoli, district of Bengazi, North Africa; and still a little later in Western Arabia. The scene of the outbreak in Mesopotamia is memorable in ancient history as being within the confines of Babylonia, not far distant from the ruins of the great city. Here, among the Afij Arabs, who occupy the first of the great marshes which exist on the east bank of the river, and of which a series extends to the junction of the Euphrates with the Tigris, the disease appeared. Spreading from the marshes, it extended along both banks of the river, attacking the different towns and villages as high as Hiliah. Traveling westward, also, it passed the marshes of Hindieh, on the west bank of the river, the scene of the outbreak of plague in 1867, and attacked the two cities, sacred to the Shiite Mahomedans, of Meshed Ali (Nedjef) and Meshed Hussein (Kerbela) on the border of the great Arabian desert. It is estimated that this outbreak carried off 4,000 persons out of a population of 90,000. The outbreak of plague in North Africa was first recognized at Merdj, a village twenty hours distant from Bengazi. It is stated to have appeared first among a Bedouin tribe encamped near the village, then to have extended to the village, and to have subsequently spread among the tribes inhabiting the district. Here the outbreak was preceded by and occurred at a time of famine, and when the country about Merdj had been converted into a vast marsh by a protracted rain. A French physician, Dr. Laval, unfortunately contracted plague in investigating this occurrence, and died from the disease. The outbreak is now reported to have ceased, but the loss of life it has caused is not yet known. The district of Bengazi was affected with plague in 1851. The scene of the outbreak of the plague in Western Arabia is in the mountainous Assyr district, North Yemen. Here, also, it is stated, in a recent report, that the disease is at an end. This outbreak would seem, so far as is yet known, to have been very circumscribed, but of its nature no doubt appears to be entertained. Plague, it is said, had not been known in Yemen since 1816 until this appearance.

The probable cessation of cholera on the Continent relieves Europe from an incubus which has been weighing heavily upon it for ten years. The disease has been uninterruptedly present in Europe ever since its extension from Egypt to the southern coast of the Continent in 1865.

Port Sanitary Authorities are increasing in number and importance, and their influence in checking the importation and perpetuation of epidemic diseases is being acknowledged and appreciated. The systematic inspection of vessels immediately after arrival, the removal and isolation of the sick, the examination of the drinking water, the closet accommodation and state of bilges, the disinfection of the clothing, the inspection of foul cargoes, and other miscellaneous duties, are found to be within the scope of work of a port medical officer. At the beginning of the current month a large body of Russian and German emigrants imported small-pox into the Thames, and, although the sick and suspected were at once separated from the healthy, the latter proved to be infected, and took the disease to Liverpool, where, however, it was successfully circumvented by the local authorities. The half-yearly reports issued by the Corporation of London, as sanitary authority of the port, show the large amount of sanitary work that has to be done on this as on other rivers. Scurvy has exhibited a slight tendency to increase in our mercantile marine. Seven official inquiries have been held by the medical officer of the Board of Trade during the year on account of outbreaks of this disease; and in one of these the master of the ship was prosecuted and fined for non-compliance with the sanitary clauses of the Merchant Shipping Act. Some progress has been made in the ventilation of ships of all classes; and much attention has been directed to this important subject, chiefly with reference to ironclads and to school ships.

The Sanitary Commission during the year has issued twenty-one reports—viz., four on the causes of certain outbreaks of fever, six on the drainage and general sanitary condition of important towns and districts, and eleven on sanitary matters of general interest, such as inquiries into the condition of the dwellings of the poor, and the influence of chemical manufactures on health, etc. The outbreak of typhoid fever at Cambridge was the subject of our first report. The disease appeared late in the autumn of last year, and, after slowly making its way along a certain line of drainage, culminated in a severe outbreak in the new buildings of Cains College. We attributed the spread of the disease to the abominable condition of the town drains, and to the percolation of sewage matter into the wells; and suggested that the cause of the outbreak at Cains College would be found in some defect in the main drain, by which contaminated excretory matter or sewer gas had found access to the College buildings. Dr. Buchanan some weeks later found that a portion of fæcal matter had been sucked up from this drain, and had contaminated the water supply of that part of the College where the cases occurred. Our inquiry into the health and management of the children at the Brentford Union Work-house, led us to the conclusion that the state of our work-houses is rapidly reverting to the status quo ante bellum 1866, and served to increase a growing feeling in favor of boarding out pauper children. The new drainage works at Brighton were the subject of a lengthy report.

Although we have no such important contribution to chronicle this year as that of Surgeon Lewis' very remarkable discovery of the presence of filaria circulating in the human blood, the members of the Indian and British medical services have contributed their quota to the scientific work of the year. The Ninth Annual Report of the Sanitary Commissioner with the Government of India contains a remarkable paper, illustrated by some fifty-nine colored drawings, on the floating bodies in the air of that country, by Dr. D. D. Cunningham, of the Indian Medical Service, who has been employed under Government, in conjunction with Dr. Lewis, of the British army, in the investigation of cholera in India. Dr. Cunningham has also, in addition, made many miscellaneous æroscopic observations in connection with the presence, development, and influence of bacteroid and other bodies found in the air of certain localities, and in that of sewers, from which he draws many interesting conclusions, one of which is that no connection can be traced between the number of bacteria, spores, etc., present in the air, and the occurrence of disease, nor between the presence or abundance of any special form or forms of cells, and the prevalence of diarrhœa, dysentery, cholera, ague, etc. Dr. Vandyke Carter has prosecuted and published his researches into the affection known as mycetoma, or fungus-disease of India. A Commission composed of the medical authorities at Calcutta have instituted an exhaustive series of experiments into the nature and effects of Indian snake-poison, as compared with that of Australian snakes—the Australian tiger-snake, for example,—which have established the correctness of the results previously arrived at by Dr. Fayrer, and proved the inefficacy of the ammonia treatment of snake-bite.

The Ashantee war gave several naval medical officers opportunities of distinguishing themselves, and these received their rewards in early and well-deserved promotion.

In surgery we have to note the issue of a third edition of Dr. Pirrie's book, and a work embodying the observations and lectures of Mr. Cæsar Hawkins. We must not omit to mention with satisfaction the papers in the "Lancet" on Hospital Construction, by Dr. Sutherland and Captain Douglas Galton. Our tribute to the merit of Dr. Barne's work on the Clinical History of the Medical and Surgical Diseases of Women has been fully endorsed by the Profession. A new edition of Meigs' and Pepper's Diseases of Children, and Dr. West's well-known work on the same subject, deserve a passing word. We reviewed at much length a number of works on the Anatomy and Diseases of the Ear, by Politzer, Hinton, Turnbull, Allen, and others. A new edition of Dunglison's Dictionary of Medical Science, large and valuable as it undoubtedly is, would still be benefited by careful revision. In Mental Physiology, Dr. Carpenter's "Principles" deserves to be named as an important contribution to that subject. In Sanitary Science and Army Hygiene, we have had many works in connection with the late Franco-Prussian war, Lex's and Roth's contributions, and Dr. Guy's Lectures on the Sanitary Aspects of War. Pettigrew on Animal Locomotion has excited some controversy.

The Hospital Sunday movement grows and extends everywhere. In London, auspiciously begun in 1873, it expanded in the present year, both as regards the sum raised and the number of contributing congregations.

The roll-call reveals many gaps in the professional ranks. Early in the year, Mr. Wormald, senior member of St. Bartholomew's surgical staff, died at a ripe age; and Mr. Thompson Dickson closed prematurely a promising career at Guy's. Dr. Forbes Winslow had not quite survived his vigorous and devoted energies in medico-psychology when he too was summoned to "the majority." Medical physics owes much to Dr. Neil Arnott, medical diagnosis to Dr. A. Kilgour, and the limitation of criminal responsibility in courts of law to Dr. Winslow. The Bombay army boasts of few abler medical officers than Dr. John Maclennan, who discharged with equal promptitude and effect duties apparently diametrically opposed; but a yet severer loss than his was that of

Cruveilhier, the great anatomist, physiologist and surgeon, a worthy disciple of Dupuytren, and long one of the brightest ornaments of the great Parisian school. "Literary labor," says his compatriot and eulogist, M. Bardinet (himself just numbered with the dead), "hospital practice, patients at home and abroad, faculty engagements, lecturing, and so on, absorbed eighteen or nineteen of the twenty-four hours, and this herculean work was carried on for many years. Rarely has a man of science been so thoroughly kind and considerate to his patients; rarely have the latter more warmly reciprocated a welcome so sympathizing." Truly "his works do follow him," for few anatomists of the century continue to fill a greater or more honored place on the book-shelves of the learned. About the same time, England—and, indeed, the whole civilized world—began reluctantly to persuade themselves that the mightiest missionary explorer since Las Casas or Xavier had died in David Livingstone—died indomitable to the last, after a life which more than any other, realized Arnauld's noble rejoinder when entreated to rest—"Rest! we shall rest through eternity!" The arrival of his mortal part on our shores enabled Sir William Fergusson to identify it by the recognition of the false joint which Livingstone had asked Sir William to examine in London some seventeen years before. The honor of a tomb in England's Walhalla, Westminster Abbey, was accorded to him; but his monument—

> "ære perennius
> Regalique situ Pyramidum altius,"—

will be that disenshrouded Africa with its slavery suppressed and its moral and religious darkness replaced by Christian science.

Dr. Anstie's death was the cause to us of almost domestic grief, and threw our pages, which he had so much enriched, into mourning. We need not recount again his virtues,—

> "Since Heaven, what praise we offer to his name,
> Hath rendered too authentic by its choice."

We must here leave our attempt to exhaust the history of medical labor. We can only, as busy men, snatch fragments from the accumulating pile of the stately building of medical science and art. We should especially like to have attempted to do some justice to our foreign brethren, and to our American—we were going to say—fellow-countrymen ; but we will content ourselves by referring to our constant attempt to represent in our columns the points of all their scientific and practical work. Witness the Foreign Gleanings, the letters of our Paris Correspondent, and our frequent leaders on physiological or pathological questions. As regards our American friends, what English surgeon did not feel proud as he read Mr. Erichsen's address, winding up with that accomplishment of Dr. Sayre, the removal of the head of the femur fifty-two times, bearing the same relation to Mr. Syme's excision of the elbow that the Reform Bill of 1867 bore to that of 1832. Medicine is becoming more and more an affair of public interest. "Internationalism" has no dread to suggest when it represents the discussion of sanitary questions at Vienna or physiological questions at Kasan. Be it ours in the future not only to hold high the domestic and social interests of the Profession, but also to recognize medical labors everywhere, whether applied to minute and impalpable questions in pathology, or to those great questions which affect the health and happiness of communities.

